# Duration of luteal support (DOLS) with progesterone pessaries to improve the success rates in assisted conception: study protocol for a randomized controlled trial

**DOI:** 10.1186/1745-6215-13-118

**Published:** 2012-07-26

**Authors:** Rafet Gazvani, Richard Russell, Yasmin Sajjad, Zarko Alfirevic

**Affiliations:** 1 Consultant Gynaecologist, Liverpool Women’s Hospital, Crown Street, Liverpool, L8 7SS, UK; 2 Subspecialist Trainee, Liverpool Women’s Hospital, Crown Street, Liverpool, L8 7SS, UK; 3 Consultant Gynaecologist, St Mary’s Hospital, Oxford Road, M13 9WL, Manchester, UK; 4 Head of Department, University of Liverpool, Crown Street, L8 7SS, Liverpool, UK

**Keywords:** IVF, Luteal phase, Progesterone, Support

## Abstract

**Background:**

Luteal support with progesterone is necessary for successful implantation of the embryo following egg collection and embryo transfer in an in-vitro fertilization (IVF) cycle. Progesterone has been used for as little as 2 weeks and for as long as 12 weeks of gestation. The optimal length of treatment is unresolved at present and it remains unclear how long to treat women receiving luteal supplementation.

**Design:**

The trial is a prospective, randomized, double-blind, placebo-controlled trial to investigate the effect of the duration of luteal support with progesterone in IVF cycles. Following 2 weeks standard treatment and a positive biochemical pregnancy test, this randomized control trial will allocate women to a supplementary 8 weeks treatment with vaginal progesterone or 8 weeks placebo. Further studies would be required to investigate whether additional supplementation with progesterone is beneficial in early pregnancy.

**Discussion:**

Currently at the Hewitt Centre, approximately 32.5% of women have a positive biochemical pregnancy test 2 weeks after embryo transfer. It is this population that is eligible for trial entry and randomization. Once the patient has confirmed a positive urinary pregnancy test they will be invited to join the trial. Once the consent form has been completed by the patient a trial prescription sheet will be sent to pharmacy with a stated collection time. The patient can then be randomized and the drugs dispensed according to pharmacy protocol. A blood sample will then be drawn for measurement of baseline hormone levels (progesterone, estradiol, free beta-human chorionic gonadotrophin, pregnancy-associated plasma protein-A, Activin A, Inhibin A and Inhibin B). The primary outcome measure is the proportion of all randomized women that continue successfully to a viable pregnancy (at least one fetus with fetal heart rate >100 beats/minute) on transabdominal/transvaginal ultrasound at 10 weeks post embryo transfer/12 weeks gestation (that is at the end of 8 weeks supplementary trial treatment).

**Trial registration:**

ISRCTN05696887

## Background

The aspiration of the granulosa cells that surround the oocyte and the use of gonadotrophin releasing hormone agonists (GnRHa) during assisted reproduction can interfere with the production of progesterone during the luteal phase. Progesterone is necessary for successful implantation of the embryo. Luteal phase support is therefore a requirement for optimal outcome following egg collection and embryo transfer in an in-vitro fertilization (IVF) cycle [1]. It has been shown that ovarian stimulation during IVF treatment, particularly in cycles incorporating GnRHa, leads to an endocrinological disturbance during the luteal phase [2,3] by impairing corpus luteum function, resulting in luteal phase insufficiency [2,3]. This is thought to be due to disturbance of pituitary function possibly in conjunction with an elevated estradiol concentration following ovarian stimulation [4].

Additionally, aspiration of the granulosa cells that surround the oocyte and the use of GnRHa during assisted reproduction treatment interferes with the production of progesterone during the luteal phase. The purpose of GnRHa in assisted reproduction is to prevent a premature surge of luteinizing hormone (LH), and this is associated with persistent blockage of LH output for 10 days after discontinuing administration [5,6]. This prolonged administration of GnRHa may also affect ovarian steroidogenesis and consequently the ability of the corpus luteum to produce progesterone [7].

### Luteal phase support and pregnancy

Progesterone is a licensed (Cyclogest®, Actavis, Devon, UK, PL00142/0508; Gestone®, Nordic, Reading UK, PL05827/0013; Crinone®, Merck Serono, Middlesex, UK, PL 03400/0081) progestational steroid necessary for the successful implantation of the embryo. Ovarian stimulation results in multiple follicular maturation, and hence in supraphysiological concentrations of estradiol and progesterone during the early luteal phase. However, these elevated steroid concentrations may also impair uterine receptivity. Furthermore, strong responses to ovarian stimulation can result in increased uterine motility at the time of embryo transfer [8] and this has been associated with a decreased pregnancy rate [9]. Luteal phase support has been shown by a meta-analysis of prospective randomized studies to be beneficial in establishing a pregnancy after IVF [10].

Both human chorionic gonadotrophin (hCG), which stimulates steroid production in the corpus luteum, and progesterone administration in the luteal phase are effective and significantly improve the clinical pregnancy rate [7,10,11]. However, hCG treatment has been shown to be associated with an increased risk of ovarian hyperstimulation syndrome [12]. Therefore an alternative approach to luteal support is supplementation with exogenous progesterone. A meta-analysis of randomized trials of luteal support showed that both hCG and progesterone supplementation produced significantly higher pregnancy rates then did placebo treatment.

### Administration of progesterone

Progesterone can be administered by several routes. The oral, intramuscular (IM) and vaginal routes have been chosen frequently in the past. The oral route is ineffective, since progesterone has a low oral bioavailability (<10%), and is associated with a high rate of metabolites which may result in side effects such as a somnolent effect comparable to that of benzodiazepine [13]. Furthermore, several randomized trials have shown oral progesterone to be associated with significantly lower implantation and pregnancy rates, and higher miscarriage rates, or both, compared with IM or vaginal administration [14,15].

IM administration of progesterone provides very high serum levels of progesterone and is associated with normal endometrial development and pregnancy rates [16-18]. However IM injection is uncomfortable for the patient and can produce serious side effects, such as injury to the sciatic nerve with impairment of sensory or motor function of the lower extremity [19] as well as a reaction to the oil vehicle.

Vaginal administration of progesterone offers a number of advantages in terms of patient convenience and tolerability. Vaginal administration has shown to be at least as effective as IM administration, and significantly more effective then oral treatment [16-18]. This is due to the first uterine pass effect, whereby progesterone reaches the uterus directly after vaginal administration without first passing through the liver [20-22]. Endometrial progesterone concentration reaches a steady state within 5 h after vaginal administration [20].

### Duration of luteal support

The next question arises as to the duration of luteal supplementation. It has been used for as little as 2 weeks and for as long as 12 weeks of gestation. The optimal length of treatment is unresolved at present and it remains unclear how long to treat women receiving luteal supplementation. It is necessary to determine when progesterone supplementation can be withdrawn without compromising the success rates of IVF treatments.

At the beginning of pregnancy, the endometrium and embryo continue to receive progesterone from the corpora lutea. The luteoplacental shift, when progesterone production is taken over by the developing placenta, does not take place until about weeks 8 to 10 of pregnancy [4]. Studies have shown that the level of progesterone is 75% in week 6 of pregnancy and falls to 50% and 25% in week 10 and week 15 of pregnancy, respectively [23]. There is a marked increase in the placental production of progesterone after week 8 of pregnancy, and therefore the luteoplacental shift begins at this point in time [24]. So far, one prospective randomized study [24] has reported that administration of progesterone up to the time clinical pregnancy is detected significantly reduces the rate of subclinical miscarriage, whilst continued administration beyond this time produced no apparent benefit. In this particular study the stimulation protocol was heterogeneous and the number of patients studied was small. These outcomes require confirmation in larger studies.

### Objective

We want to conduct a prospective, randomized, double-blind study to investigate the effect of the duration of luteal support with progesterone in IVF cycles. Following 2 weeks standard treatment and a positive biochemical pregnancy test, this randomized control trial will allocate women to a supplementary 8 weeks treatment with vaginal progesterone or 8 weeks placebo. Further studies would be required to investigate whether additional supplementation with progesterone is beneficial in early pregnancy.

## Methods and design

### Patient selection

Ethical approval will be obtained from the Liverpool Research Ethics Committee and the Liverpool Women’s National Health Service (NHS) Foundation Trust Research and Development (R&D) Committee before the trial starts. Follow-up of these patients will occur in a range of hospitals according to standard practice and are hence exempt from site-specific assessment. An information leaflet will be given to all women and written informed consent will be obtained from those agreeing to take part. The trial will be conducted according to good clinical practice (GCP) guidelines, the NHS Research Governance Framework and the Helsinki Declaration.

### Stimulation protocols

Study participants will receive ovarian stimulation according to standardized unit protocol. This is standard treatment and is the same for both trial and non-trial participants and is independent of the trial protocol. This involves luteal phase GnRHa downregulation. In accordance with this protocol, 500 μg Buserelin acetate is administered daily by subcutaneous injection from the mid-luteal phase. This dose is continued until ovarian suppression is achieved, which is ascertained with transvaginal ultrasound (endometrial thickness less than 5 mm and no ovarian follicles more than 6 mm). Ovarian stimulation is then commenced using human menopausal gonadotrophin (hMG) (Serono, Middlesex, UK), the initial dose being dependent on the patient’s age, body mass index, the basal follicle stimulating hormone (FSH) values and any prior responses to ovarian stimulation. The ovarian response is assessed on day 10 of the treatment by a transvaginal scan. When at least one follicle has reached a diameter of 17 mm and two follicles have reached a diameter of 16 mm (unit protocol), hCG (Profasi; Serono) 5000 IU is given and oocyte retrieval is performed 36 h later by vaginal ultrasound-guided follicle aspiration.

### Luteal support

Luteal support is initiated after egg retrieval. The women are asked to start using 400 mg progesterone (Cyclogest®; Actavis) pessaries a day before the embryo transfer rectally (to keep the vagina clean for the procedure). Embryo transfer is performed 48 h after the egg retrieval. After the embryo transfer, the pessaries are either used rectally or per vaginum depending on the patients’ choice. At present in our unit the luteal support is continued until the pregnancy test is performed which is 2 weeks after the embryo transfer whilst some units continue the luteal support for up to 10 weeks after the positive pregnancy test, in other words until 12 weeks of gestation.

### Trial design

#### Trial eligibility

All women presenting to the Hewitt Centre for Reproductive Medicine, Liverpool Women’s NHS Foundation Trust, for assisted conception with a positive biochemical pregnancy test at 2 weeks post-embryo transfer are eligible to enter the trial.

#### Recruitment

The Reproductive Medicine Unit at the Liverpool Women’s NHS Foundation Trust holds a patient information evening once a month for couples about to undergo assisted conception treatment. It is during this visit that the trial will be fully explained to the women (and their partners) so they have a preliminary idea of the study.

They then return to the clinic to commence treatment approximately 5 to 6 weeks later. They are given a further opportunity to ask any questions regarding the trial and a patient information leaflet is given to them to keep for reference. The patient information leaflet gives the contact details of the Chief Investigator should the couple wish to ask any questions.

Actual treatment is initiated 2 to 3 weeks after the initial clinic appointment. After this they are given an appointment to collect their treatment drugs (note: baseline hormone levels are measured after the patient information evening). It is at this point that their IVF drugs are dispensed. This is the standard procedure.

The treatment begins with downregulation on day 23 of their menstrual cycle when they use Buserelin acetate injection/nasal spray for 3 weeks. At the end of this treatment they have a scan. Ovarian stimulation is then commenced and a scan is performed on day 10 of ovarian stimulation to assess the response. If the response is appropriate, their eggs are collected on days 12 or 13 depending on the response of the ovaries. Two days after the eggs are collected the embryos are transferred. The women are instructed to start taking the progesterone pessaries the night before the embryo transfer (400 mg pessary once). After the embryo transfer they continue with the pessaries 400 mg twice a day until the day of the pregnancy test. At this stage they will have a further opportunity to ask questions regarding the study should they wish to. Patients will be handed a Duration Of Luteal Support (DOLS) trial patient information leaflet and consent form that has been completed by the researcher. They will be asked to retain this with them and complete the relevant sections once agreement has been made to join the trial following a positive pregnancy test (see notes below). A urinary pregnancy test is arranged for 14 days after the embryo transfer. This is standard practice for women undergoing assisted conception on the Reproductive Medicine Unit.

Currently at the Hewitt Centre, approximately 32.5% of women have a positive biochemical pregnancy test 2 weeks after embryo transfer. It is this population that is eligible for trial entry and randomization. Once the patient has confirmed a positive urinary pregnancy test they will be invited to join the trial. Eligibility will be confirmed verbally over the telephone according to the trial entry protocol. The patient will then be asked to sign the consent form that they have with them at home and return it to the Hewitt Centre within 24 h. Once the consent form has been completed by the patient, a trial prescription sheet will be sent to the pharmacy with a stated collection time. The patient can then be randomized and the drugs dispensed according to pharmacy protocol. A blood sample will then be drawn for measurement of baseline hormone levels (progesterone, estradiol, free beta-hCG, pregnancy-associated plasma protein-A (PAPP-A), Activin A, Inhibin A and Inhibin B).

The patient will then be given the drug pack that has been prepared by the pharmacy. This drug pack will contain either the placebo pessaries or the progesterone pessaries. They will continue to take these for a further 8 weeks (in other words, up to 10 weeks post-embryo transfer, which is up to the 12 weeks gestation calculated by the last menstrual period).

Inclusion criteria:

1. Biochemical pregnancy confirmed by urinary pregnancy test. If positive then they will be eligible to enter the trial.

2. Progesterone treatment (2 weeks) completed up to the day of the pregnancy test.

3. Patients treated at Hewitt centre only.

4. Only those patients who have been treated with the long-stimulation protocol.

Exclusion criteria

1. All women who are undergoing frozen embryo transfer.

2. Any women not willing to take part will be excluded.

3. All diabetic, epileptic and hypertensive patients on treatment.

4. Poorly controlled asthmatics having had more than one hospital admission in the last year.

5. Patients with renal and cardiac dysfunction (under the care of nephrologist and cardiologist, respectively).

6. Breast cancer patients.

7. Severe liver impairment/jaundice.

8. Previous history of thromboembolism.

9. Transport centre patients (that is, egg collection outside the main hospital).

Five weeks after the embryo transfer (that is, 7 weeks gestation by last menstrual period) the women will have a first pregnancy scan (this is a standard protocol for all our unit patients). This scan will check for the viability of pregnancy in the form of positive heart beat pulsations. At this point a pelvic Doppler will also be performed to determine the blood velocity in the uterine arteries (the main vessels which supply blood to the womb). This will enable us to compare the blood flow between the two treatment groups.

At this appointment, a further 15 ml venous blood sample will be taken to check for progesterone, estradiol, free beta-hCG, Inhibin A, Inhibin B, PAPP-A, and Activin A. Patients with a viable pregnancy as confirmed by an ultrasound will continue with the pessaries for up to 10 weeks post-embryo transfer (that is, 12 weeks gestation).as explained below:

Patients with a viable pregnancy at this point will be informed of the various antenatal screening methods available to them (that is, triple test, quadruple test, nuchal translucency, etc.) as per unit standard practice.

At the end of the week 10 post-embryo transfer (that is, 12 weeks gestation) the women will be asked to attend the unit for a further pregnancy scan to determine the viability of the pregnancy and record the fetal heart rate (FHR). At this appointment the second uterine artery Doppler is performed to compare the blood velocity between the two groups. A further 15ml venous blood will be taken to check for progesterone, PAPP-A, estradiol, free B-hCG, Inhibin A, Inhibin B and Activin A.

At approximately 34 weeks gestation a courtesy phone call will be made to the patient to enquire how the pregnancy is proceeding. This will be undertaken by the medical/research team heading the trial.

Following delivery, birth details and neonatal outcome will be observed.

Treatment allocation

1. Following a positive biochemical urinary pregnancy test, the patient will be asked to attend the reproductive unit if not present already. After eligibility has been confirmed the patient will be asked to sign the trial consent form. This will be done by a member of the Hewitt Centre Staff who is trial trained and GCP compliant.

2. Pharmacy will be alerted that a treatment pack (either active ingredient or placebo) will need to be made available for collection. The pharmacy will be given the patients’ age (less than 37 years old/greater than 37 years old) so that the appropriate randomization sequence is accessed and drug pack dispensed.

3. The treatment pack will be collected by a member of staff from the reproductive medicine unit along with a treatment pack number. The treatment pack number will be entered with the patients’ details in the trial pharmacy log book. These details will be completed, signed and dated by the Pharmacist for verification purposes.

4. The treatment pack will be given directly to the patient by a member of the reproductive unit, along with confirming instructions for use. Patient details and treatment pack number will also be entered in to the trial register held on the reproductive medicine unit.

Each study participant will be given a blister pack which contains either 400 mg progesterone pessaries or identical appearing pessaries of placebo. The drug and placebo are provided by Actavis, which had no involvement in study design, data collection, data handling, data analysis, study interpretation, drafting of manuscripts or decisions to publish. Each patient pack will contain 140 pessaries. This supply will enable enough pessaries to last for 8 weeks (that is, until 10 weeks post-embryo transfer/12 weeks gestation by last menstrual period), and also enough additional pessaries to account for natural wastage as often occurs during treatment. Natural wastage refers to the process whereby the treatment pessary is lost due to premature expulsion from either the vagina or rectum prior to the optimum application procedure time.

Excess pessaries that are not used by the patient during the trial period will be collected from the patient at their 12-week follow-up scan. These drugs will be reconciled with pharmacy and disposed of according to hospital policy.

There is a separate randomization sequence for patients stratified less than 37 years of age and patients older than 37 years of age. The randomization sequences were prepared for the trial by Anna Hart (trial statistician) using random sized blocks (2, 4 and 6) using the statistical package available at http://www.randomization.com . Two randomization lists have been prepared. The first list for enrolled women greater than 37 years of age where all ID prefixes begin with a “1” (total 180, 90 per group) and the second list for patients younger than 37 years old where all the ID numbers begin with a “2” (total 360, 180 per group). Trial investigators are to be kept blind to all randomization sequences.

Patients hospital notes are marked with a sticker labeled “This patient is participating in the DOLS study”.

Patients are informed of the date and time of their subsequent second scan which is to be performed 3 weeks following positive urinary pregnancy test (that is, 5 weeks post-embryo transfer/7 weeks gestation).

The investigators, participants and clinicians are unaware of the treatment assignments. Although treatments are blinded, the head pharmacist and the on-call pharmacist will have access to the randomization codes should the need to break the code arise.

Information on patient characteristics, demographics and treatment are transcribed from the hospital case notes to the case report form.

## Discussion

### Measure of outcomes

Primary outcome measure:The primary outcome measure is the proportion of all randomized women that continue successfully to a viable pregnancy (at least one fetus with FHR >100 beats/minute) on transabdominal/transvaginal ultrasound at 10 weeks post-embryo transfer/12 weeks gestation (that is, at the end of 8 weeks supplementary trial treatment). The following clinical estimates are available. The success rate at the biochemical testing stage is approximately 32.5%. Currently, without further progesterone supplementation, 80% of these women will carry on successfully with a viable pregnancy (25.8% of clinical pregnancy rate) whilst 20% of these women will miscarry. We anticipate that an increase of 10% from the biochemical to the clinical pregnancy rate would be clinically important and therefore justify an additional 8 weeks supplementation with progesterone, in other words, a reduction of 10% in the miscarriage rate.

Secondary outcomes:

1. Viable pregnancy (at least one fetus with FHR > 100 beats/minute) on transvaginal ultrasound at 5 weeks post-embryo transfer/7 weeks gestation (that is, at the end of 3 weeks supplementary trial treatment).

2. Relationship of serum markers (estradiol, progesterone, Inhibin A, Inhibin B, Actavin A, free beta-hCG and PAPP-A) taken at 5 and 10 weeks post-embryo transfer with treatment allocation, primary outcome and secondary outcomes.

3. A pelvic Doppler will be performed to determine if the blood velocity in the uterine artery is different in the two groups. This will be performed at 5 and 10 weeks post-embryo transfer.

4. Side effects

A visual analogue score relating to the following side effects will be completed at both 5 weeks and 10 weeks post-embryo transfer: nausea, bloating, vaginal discharge and vaginal irritation.

5. Antenatal Down’s Screening Outcomes

Antenatal Down’s screening results for all patients will be monitored, including individual serum quantification for double, triple, quadruple screening and nuchal translucency.

6. Neonatal outcomes

Outcome data regarding the child will be collected at delivery. A standard neonatal examination will be conducted. Women will be asked to inform the study team of the outcome of the routine neonatal examination. Excessive androgenization would be an important adverse outcome of supplementary progesterone and this would be detected by routine neonatal examination. Long-term follow-up of the child may also be conducted as a separate study. Women will be asked whether they consent to being contacted in future about long-term follow-up studies.Some neonatal and other pregnancy outcomes will be captured in Trusts that do not recruit to this trial. We will identify a local contact and R&D contact in each Trust in order to obtain the necessary approvals. At each site that will contribute data to the outcomes clinicians will be following their routine clinical practice. Consent to participate in the study will not be sought at sites that contribute to data collection about outcomes and study medication will not be administered at these sites. Thus, the sites that contribute to data collection about outcomes will be exempt from site-specific assessment as part of approval by the Research Ethics Committee.

7. Compliance data

Patient compliance with using pessaries is recorded at each visit. A secondary analysis may look at the effect of compliance on the primary outcome.

#### Data collection

Information will be collected at the following times:

Trial entry (from clinical notes)

At 5 weeks post-embryo transfer – following a fetal ultrasound scan to determine viability of the pregnancy and uterine artery Doppler parameters (from clinical notes).

At 10 weeks post-embryo transfer – following fetal ultrasound scan to determine the viability of the pregnancy and uterine artery Doppler parameters (from clinical notes).

Serum results will be returned in batch form at the end of the trial directly to the case report file.

Antenatal screening results (Each patient is given a standard form with self-addressed stamped envelope at the second ultrasound scan to be completed following antenatal screening). Data will be entered directly to the case report file.

The outcome of the pregnancy will be recorded after delivery. Each patient is given a standard letter which is to be completed after delivery. Data can also be obtained from the reproductive centre database (HFEA requirement). Information regarding delivery will also be recorded in the clinical case notes.

A neonatal assessment will be performed shortly after delivery by a member of the neonatal team in the delivery hospital. Each patient is given a standard form with self-addressed stamped envelope at the second ultrasound scan which is to be returned with the delivery details.

There may be a long-term follow-up of all the live births in the trial.

#### Safety considerations

Before recruitment into the trial women will be assessed in terms of their safety for entering the trial during screening via the trial’s entry form. Women in whom progesterone is contraindicated, such as those with liver dysfunction, or women with conditions which may worsen with fluid retention, such as epilepsy, hypertension, asthma, diabetes, cardiac or renal dysfunction or previous history of venous thrombo-embolism, will be excluded from the trial.

As progesterone is a natural hormone, it is not expected to cause serious adverse events in women carefully selected to avoid contraindications and to minimize.

A serious adverse event (SAE) (may or may not be considered related to the Progesterone/placebo pessaries), which results in death, is life threatening, requires hospitalisation or prolongation of inpatient hospitalisation, or results in persistent of significant disability or incapacity.

A serious adverse reaction (SAR) is any adverse reaction (caused by the progesterone/placebo pessaries) which results in death, is life threatening, requires hospitalization or prolongation of existing inpatient hospitalization, or results in persistent or significant disability or incapacity. Examples that may occur in the DOLS trial are jaundice and anaphylactoid reactions.

A suspected unexpected serious adverse reaction (SUSAR) is an adverse reaction which is both unexpected and serious.

SARs or SUSARs will be recorded and reported using the SAE report form (see Appendix C). In the event of a SUSAR, the form will be completed by the trial investigators and given directly to the R&D Department of the Trust within 24 h. The R&D Department, on behalf of the Sponsor, will inform the Chair of the Data Monitoring Committee (DMC) immediately, and the competent authorities within 7 days if the event is life threatening or 15 days if not.

#### Withdrawals

It is expected that the number of withdrawals will be very low due to the highly motivated nature of our patient population.

The DOLS trial is a pragmatic study, and therefore data should be analyzed as intention-to-treat; in other words, analyzing data according to the group to which women were randomized, whether or not they actually comply with the treatment. Stopping treatment does not constitute withdrawal from the study.

#### Analysis

The analysis of this trial will be by ‘intention-to-treat’; therefore, women will be analyzed by the groups into which they were randomized regardless of what intervention they received.

A two group chi-squared test with a 5% two-sided significance level will have 80% power to detect the difference between a control group proportion 80% and a treatment group proportion 90% (odds ratio of 2.151) when the sample size in each group is 210.

Given that the method of analysis is logistic regression with age as a factor, patients will be stratified into two groups; those less than 37 years of age and those older than or equal to 37 years of age. It is recognized that age is an important variable when discussing success rates in assisted conception. To account for this, stratification was employed to optimize the distribution of patients of all ages. A suitable sample size would be 230 per group.

It is anticipated that the drop-outs would be minimal. We aim to recruit a total of 460 patients but we anticipate randomizing no more than 480 women to achieve.

The statistician will remain blinded to the actual identity of the groups for as much analysis as possible.

#### Primary outcome

The primary outcome (proportion of viable pregnancies) will be summarized with a relative risk and 95% confidence interval. The primary analysis will be logistic regression taking into account the age of the woman (that is, the stratification variable).

If other important prognostic factors are imbalanced between the two groups the robustness of the findings will be investigated by including other covariates in the logistic regression.

Although it is anticipated that withdrawal rates will be low, there may be missing values. A range of clinically plausible scenarios will be used to impute missing values in order to test the robustness of the findings. Details of this will be approved by Data Monitoring Committee (DMC) once the magnitude of the problem is known. The statistician may need to be unblinded for these analyses in order to carry out realistic, conservative analyses.

The effect of compliance (as a suitably coded factor) will be investigated by testing the interaction between treatment group and compliance.

#### Secondary outcomes

These will be exploratory analyses producing 95% confidence intervals. They will be unadjusted for multiple analyses. The interpretation will take multiple testing and the consequent likelihood of type I errors into account qualitatively.

Continuous outcomes will be analyzed by analysis of covariance and dichotomous variables by logistic regression. In a similar way to the analysis of the primary outcome, these analyses will stratify for age, and the robustness of findings will be examined by including other potentially important prognostic covariates. A range of plausible values will be imputed for missing values.

Where a large proportion of values are missing (typically >25%) only descriptive statistics will be provided.

If any variables are highly skewed, leading to doubts about the validity of the parametric analyses, non-parametric analyses will be carried out or bootstrapping will be used.

#### Demographics and other clinical variables

Other variables (for example, describing the groups at baseline or the clinical progress of the women) will be described using summary statistics only.

### Ethics

The DOLS trial has full ethical approval (REC 06/MRE08/17). Additional counseling will be offered from the research team for women with failed pregnancies.

### Data management

#### Data monitoring committee

For the trial a DMC has been established. This is independent of the trial organizers. Regular meetings are established for the duration of the trial with open and closed sessions so the DMC members can analyze the interim analysis data when required. The Trial Steering Committee (TSC) will provide the Chair of the DMC with any other analysis as is requested by the Chair.

If any other evidence from other randomized controlled trials is published during the running of the trial then the DMC will be fully informed by the TSC. With this evidence, and the interim analysis, the DMC will inform the TSC if the trial needs to stop.

All SAEs will be reported to the TSC, and in turn these will be reported to the DMC.

The unblinding of the trial is the responsibility of the Chief Investigator. Should any SUSAR be incurred the trial can be unblinded by a 24 hour telephone line within the pharmacy department. The on-call pharmacist has access to unblinding the trial.

#### Trial statistician

The trial statistician will be made aware of assignment to treatment codes (for example, A or B), but will remain blinded as to which is active ingredient or placebo. The trial statistician will be involved in the preparation of reports for DMEC. Unblinding may become necessary when invoking sensitivity analyses for missing values.

#### Data handling and storage

There is provision for dedicated personnel for the collation, storage and management of data, as provided by the Liverpool Womens’ Hospital Research and Development Trials office. Additional resources are available for data checking and double entry of data.

#### Direct access to source data/documents

The investigator(s)/institution will permit trial-related monitoring, audits, Institutional Review Board/regulatory inspections, providing direct access to source data and documents.

#### Monitoring

Monitoring will be the responsibility of the Research and Development Manager at Liverpool Womens’ NHS Foundation Trust Hospital.

#### Liability and negligence

The trust and investigators are not liable or responsible for harm encountered through participation in the trial. However, harm relating through action(s) of negligence are accountable through the trust. The National Health Service complaints mechanism is available to patients.

#### Finance

Drug packs (progesterone/placebo) were supplied by Actavis, Devon, UK.

## Trial status

The trial has completed recruiting by the 1 June 2012.

## Abbreviations

DMC: Data monitoring committee; DOLS: Duration of luteal support; FHR: Fetal heart rate; GnRHa: Gonadotrophin releasing hormone agonists; hCG: Human chorionic gonadotrophin; hMG: Human menopausal gonadotrophin; IM: Intramuscular; IVF: in-vitro fertilization; LH: Luteinizing hormone; NHS: National health service; R&D: Research and development; SAE: Serious adverse event; SAR: Serious adverse reaction; SUSAR: Suspected unexpected serious adverse reaction; TSC: Trial steering committee.

## Competing interests

The authors declare that they have no competing interests.

## Authors’ contributions

RG is the Principle investigator, author of the initial protocol, contributor to patient recruitment and author of the *Trials* journal version article of the protocol. RR is a major contributor to patient recruitment over the last three years, obtained a support grant for the trial and contributed to the *Trials* journal article version of the protocol. YS is author of the first protocol for the trial, recruited patients to the trial during the first 2 years and contributed to the *Trials* journal article version of the protocol. ZA contributed to the trial protocol and general running of the trial at all times, contributed to the writing of the original protocol and contributed to the *Trials* journal article version of the protocol. All authors read and approved the final manuscript.
